# Novel Biomarkers as Potential Predictors of Decompensated Advanced Chronic Heart Failure—Single Center Study

**DOI:** 10.3390/jcm13226866

**Published:** 2024-11-14

**Authors:** Tobias Fröhling, Dilvin Semo, Moritz Mirna, Vera Paar, Zornitsa Shomanova, Lukas J. Motloch, Andreas Rukosujew, Jürgen R. Sindermann, Michael Lichtenauer, Rudin Pistulli

**Affiliations:** 1 Department for Cardiology I, Coronary and Peripheral Vascular Disease, Heart Failure, University Hospital Münster, 48149 Münster, Germany; 2 Department of Internal Medicine II, Division of Cardiology, Paracelsus Medical University Hospital of Salzburg, 5020 Salzburg, Austria; 3 Department of Cardiothoracic Surgery, University Hospital of Münster, 48149 Münster, Germany; zornitsa.shomanova@ukmuenster.de (Z.S.); andreas.rukosujew@ukmuenster.de (A.R.); 4 Interdisciplinary Heart Failure Section, University Hospital Münster, 48149 Münster, Germany; 5 Department of Internal Medicine II, Paracelsus Medical University, 5020 Salzburg, Austria; 6 Department of Internal Medicine II, Salzkammergut Klinikum, OÖG, 4840 Vöcklabruck, Austria; 7 Department of Cardiology, Kepler University Hospital, Medical Faculty, Johannes Kepler University, 4020 Linz, Austria

**Keywords:** heart failure, biomarker, acute decompensated heart failure, HFrEF, HFmrEF

## Abstract

**Background/Objectives:** Heart failure (HF) remains a major therapeutic and diagnostic challenge nowadays. Albeit, acute decompensated HF is associated with several clinical signs such as dyspnea or edema, it remains a challenge to use easy accessible and suitable tools, such as biomarkers, to distinguish between patients at risk for an acute decompensation of their heart failure and compensated, stable HF patients. Existing biomarkers, such as natriuretic peptides or troponin, are not specific and can be elevated due to several other disease conditions, such as myocardial infarction, atrial fibrillation, or valve diseases. Therefore, the aim of this study was to analyze the predictive potential of four novel cardiovascular biomarkers—the soluble urokinase-type plasminogen activator receptor (suPAR), heart-type fatty acid binding protein (H-FABP), vascular cell adhesion molecule 1 (VCAM-1), and growth/differentiation factor 15 (GDF-15) for the detection of cardiac decompensation in patients with HF. **Methods**: In this study, 146 patients were prospectively enrolled and the serum biomarker concentrations were analyzed using Enzyme Linked Immunosorbent Assay (ELISA). We correlated the biomarker concentrations with clinical and biochemical parameters of all patients and the predictive value for detection of cardiac decompensation was assessed. **Results:** A significant increase in the levels of suPAR (1.6-fold-change, *p* < 0.0001), H-FABP (2.2-fold-change, *p* = 0.0458), VCAM-1 (1.6-fold-change, *p* < 0.0001), and GDF-15 (1.7-fold-change, *p* = 0.0009) was detected in all patients with acute decompensated HF in comparison to patients with compensated HF. Univariate logistic regression analysis revealed a significant association of biomarker plasma concentration with the risk for a cardiac decompensation (suPAR: *p* < 0.0001; VCAM-1: *p* < 0.0001, H-FABP: *p* = 0.0458; GDF-15: *p* = 0.0009). **Conclusions:** In conclusion, the investigated novel cardiovascular biomarkers suPAR, GDF-15, VCAM-1, and H-FABP could be a valuable tool to facilitate therapeutic decisions in patients with heart failure and suspicion of a cardiac decompensation. Parameters such as renal function should be taken into account. Further studies on novel biomarkers are required to find reliable, sensitive, and specific tools that will enable the early detection of patients with acute decompensation.

## 1. Introduction

With a global prevalence of 1 to 2% and its increasing risk with age, heart failure (HF) poses a major health and economic burden, due to its high mortality and morbidity despite available therapies [1,2]. According to the 2021 guidelines of the European Society of Cardiology (ESC) for the diagnosis and treatment of acute and chronic HF (CHF), acute heart failure (AHF) [3] covers all forms of disease worsening and hereby comprises various pathomechanisms. Moreover, due to unspecific symptoms in the state of acute decompensated HF and lacking established scores in clinical practice, differentiation between decompensated and compensated HF remains a major challenge. Typical symptoms, such as fatigue and shortness of breath, occur in both groups and are often insidious, so that subtle changes do not lead to early adaptation of therapy [4,5].

While biomarkers are major diagnostic tools in several diagnostic workups, to date only natriuretic peptides (such as NTproBNP) are well established in the daily assessment of HF patients. But besides being very useful in diagnosing HF and having prognostic properties [6,7,8,9], they are less reliable in differentiating between chronic and acute HF, and previous trials trying to establish their use in guiding therapy and preventing HF hospitalizations have been disappointing [10,11,12]. Since present biomarkers lack the ability to deliver reliable information on the severity of decompensated heart failure, the aim of our study was to look for alternative biomarkers, which could differentiate between stable disease (compensated heart failure) and acute progress (acute decompensated heart failure). Therefore, we evaluated the circulating biomarkers GDF-15, H-FABP, suPAR, and VCAM-1 in a single center cohort of advanced heart failure patients clinically presenting in the chronic and acute forms, thus aiming to show their sensitivity to HF decompensation [13].

## 2. Materials and Methods

### 2.1. Study Population

In this prospective, single-center study, a total of 146 non-consecutive patients were recruited. All patients provided informed consent during their hospital admission. Patients with any medical history of cancer, infections, acute kidney injury, or autoimmune disease were excluded. Patients were cared for at the interdisciplinary heart failure section of the University Hospital Münster in Germany 1 May 2019 and 30 November 2020. All patients received the diagnosis of heart failure after a diagnostic workup. The diagnosis was based upon the present guidelines of the European Society of Cardiology. The acute heart failure diagnostic workup included obtainment of patient history and clinical examination. In particular, signs of acute pulmonary edema, such as tachypnea or hypoxemia, or clinical signs of hypoperfusion (e.g., low pulse pressure, mental confusion, lactate elevation) were taken into consideration. Moreover, patients were examined for signs of peripheral edema. Investigation of pulmonary edema included lung ultrasound or chest X-ray for the detection of congestion. Echocardiography was performed. Relevant blood parameters, NTproBNP and troponin, were taken into consideration. The final diagnosis was made by two independent cardiologists. In case of any discrepancies, a third expert was involved and solved any inconsistency. The study population covered patients in the clinical state of compensated chronic heart failure (*n* = 105) and acute decompensated heart failure (*n* = 41) at time of admission. Initial assessment and clinical examination were followed by transthoracic echocardiography (TTE). Additionally, medical history and previous medication were investigated. Moreover, laboratory results were analyzed. The study was approved by the local ethics committee of the University Hospital and was conducted in accordance with the Universal Declaration of Helsinki.

### 2.2. Blood Sampling

Blood was drawn from the patients’ cubital vein and collected in serum and plasma vials. Within 20 min after blood withdrawal, samples were centrifuged at 2000× *g* at 4 °C for 20 min. Hereafter, supernatant was collected and frozen at −80 °C until further analyses were performed. Routine blood analysis performed at the time of hospital administration covered parameters such as NTproBNP (N-terminal pro-brain natriuretic peptide, pg/mL), creatinine (mg/dL), C-reactive protein (CRP, mg/dL), low-density-lipoprotein (LDL, mg/dL), and hematological parameters.

### 2.3. Biomarker Assays

Frozen serum and plasma samples were centrifuged and hereafter levels of cardiac biomarkers were measured by colorimetric detection methods. We probed for the biomarkers GDF-15 (pg/mL), H-FABP (ng/mL), suPAR (pg/mL), and VCAM-1 (ng/mL), which were chosen based on previous findings of our research group. Those biomarkers were measured with commercially available ELISA kits (DuoSet ELISA DY1678, DY523B, DY807, DY957 and DFTA00, R&D Systems, Minneapolis, MN, USA) according to the manufacturer’s instructions. In addition, 100 µL of the serum and provided standard solutions were applied to a 96-well plate after coating the 96-well plate with the provided capture antibody. The plates were incubated overnight on an orbital shaker at room temperature. After the plates were washed three times with the provided and prepared wash solution, 100 µL of the conjugate solution was added for 1 h at room temperature. After another washing step, streptavidin-horseradish peroxidase (HRP) was applied to each well for 20 min in the dark; the plates were washed again three times. Thereafter, we allowed color development by adding the streptavidin-horseradish peroxidase (HRP) solution. Lastly, optical density (OD) was measured at 450 nm using an ELISA microplate reader (iMark Microplate Absorbance Reader, Bio-Rad Laboratories, Vienna, Austria).

### 2.4. Ethics

The study conformed to the Declaration of Helsinki and is covered by an ethical approval by the local ethics committee of Münster in Germany: 2019-011-f-S, approval date of 11 March 2019. All participants provided written informed consent prior to inclusion.

### 2.5. Statistics

Statistic analyses were performed using SPSS (22.0, SPSS Inc., Chicago, IL, USA). The Kolmogorov–Smirnov test was used to assess the distribution of the data. Due to the distribution of biomarker concentration, all values were expressed as median and interquartile range (IQR). Median values between groups were compared using a Mann–Whitney U test or a Kruskal–Wallis’s test with a Dunn’s post hoc test. Correlation was carried out using Spearman’s rank correlation coefficient. ROC analysis was performed, and an optimal cut-off value was calculated using the Youden index. Areas under the curve (AUC) were compared as described by Hanley and McNeil [14]. A *p*-value below 0.05 was considered statistically significant.

## 3. Results

The baseline characteristics of all 146 patients are shown in Table 1. For this study, 105 patients were defined as compensated heart failure patients per ESC guidelines after clinical assessment. There were 41 patients who showed signs of acute decompensated heart failure. Roughly 72.4% of the compensated HF and 79.7% of the decompensated HF patients were males (*p* = 0.843). The median age was 64 years in the compensated HF and 69 years in the decompensated HF group (*p* = 0.272). Patients in the decompensated group had a significantly higher BMI than those in the compensated group (*p* < 0.001). The majority of patients were under heart failure medication with angiotensin-converting enzyme inhibitors (ACE or angiotensin-1 receptor (AT1) inhibitors (70.5 vs. 56.1%), betablockers (89.5 vs. 78%), or aldosterone-antagonist (61.9 vs. 36.6%). Additional loop diuretics were prescribed to 60% or 75.6% of the patients. SGLT-2 (sodium glucose transporter 2) inhibitors were prescribed to 1.9 and 7.3% of the patients, respectively.

**Figure 1:**
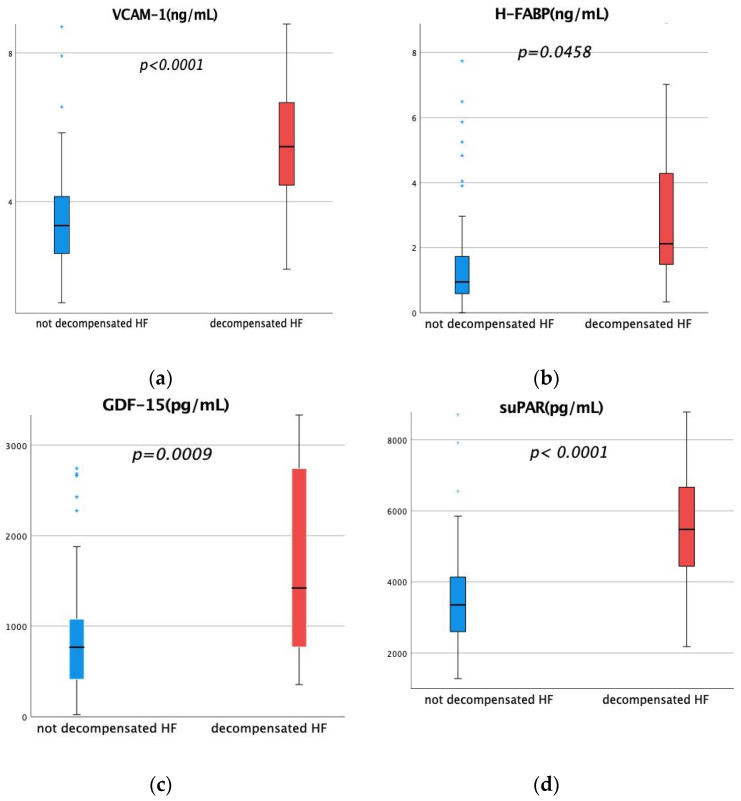
Levels of biomarker VCAM-1, H-FABP, GDF-15, and suPAR in group of compensated and acute decompensated heart failure. (**a**) Levels of VCAM-1 (ng/mL) in not decompensated and decompensated HF. (**b**) Levels of H-FABP (ng/mL) in not decompensated and decompensated HF. (**c**) Levels of GDF-15 (pg/mL) in not decompensated and decompensated HF. (**d**) Levels of suPAR (pg/mL) in not decompensated and decompensated HF. Blue asterisk sign represents values out of range.

NTproBNP levels were significantly higher in the group of acute decompensated HF (compensated HF: 1207 pg/mL (mean) and acute decompensated HF: 3798.5 pg/mL (mean); *p* < 0.001). In addition, the patients in the decompensated group appeared more often with a NYHA stadium of III (compensated HF: 40% and acute decompensated HF 63.4%; *p* < 0.008) or IV (compensated HF 2.9% and acute decompensated HF 22%; *p* = 0.007). Acute decompensated patients more often displayed pleural effusion (1.9 vs. 26.8%; *p* = 0.001) or edema (11.4 vs. 61.0%, *p* < 0.001). The distribution of heart failure entity between ischemic and non-ischemic heart failure did not show any relevant unbalance. The levels of suPAR and VCAM-1 were 1.6-fold enhanced between the group of compensated and acute decompensated HF (*p* < 0.0001). Our analysis revealed a 2.2-fold elevation of the biomarker H-FABP in acute decompensated HF *(p* = 0.0458). Moreover, serum samples of acute decompensated HF patients displayed 1.7 times higher GDF-15 levels (*p* = 0.0009) (Figure 1).

The correlations between patient characteristics and the investigated biomarkers suPAR, GDF-15, VCAM-1, and H-FABP are shown in Table 2 in a univariate logistic regression analysis. Our analysis revealed a strong association between CRP and suPAR (r = 0.464, *p* < 0.0001), and VCAM-1 (r = 0.464, *p* < 0.0001), GDF-15 (r = 0.443, *p* < 0.0001), and H-FABP (r = 0.320, *p* < 0.0001). BMI, suPAR (r = 0.232, *p* = 0.007), and VCAM-1 (r = 0.232, *p* = 0.007) appeared with a relevant correlation. After the correction for confounders by multivariate logistic regression analysis, the association of elevated biomarker levels and acute decompensated HF remained significant for suPAR (*p* = 0.0015), GDF-15 (*p* = 0.0015), and VCAM-1 (*p* = 0.0352) (Table 3).

Since the patients’ characteristics revealed a significant prescription difference in aldosterone antagonists (*p* = 0.006) and platelet inhibitors (*p* = 0.003), we performed an additional univariate regression analysis and could not detect any influence of the drug intake on the results (data shown in Supplementary Material Table S1).

**Figure 2:**
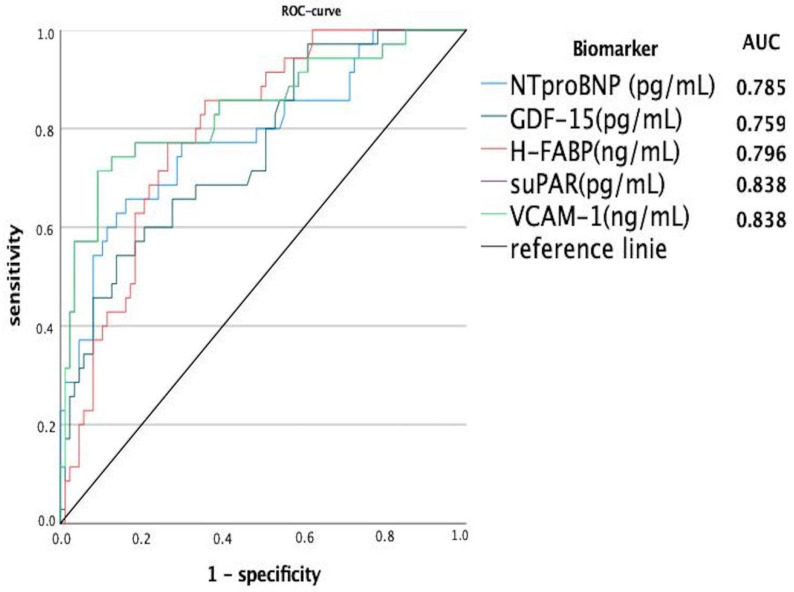
Receiver Operating Characteristic (ROC) with diagnostic potential of novel cardiac biomarkers in case of decompensated heart failure.

Furthermore, ROC analysis was performed, and AUC (area under the curve) was calculated for serum levels of suPAR, VCAM-1, and GDF-15 to test for the usability of biomarkers as a diagnostic indicator for acute decompensated HF (Figure 2). In this analysis, suPAR (0.838, 95%CI (0.750–0.926)) and VCAM-1 (0.838, 95%CI (0.750–0.926)) were identified as the important predictive biomarkers and potentially better predictors than NTproBNP. The Youden index was used to calculate optimal cut-off values for the diagnosis of decompensation (suPAR: 4774.027 pg/mL VCAM-1: 4.74027 ng/mL). AUC for GDF-15 (0.759, 95%CI (0.683–0.866)) and NTproBNP (0.785, 95%CI (0.691–0.880)) showed comparatively lower values. Sensitivity, specificity, and positive and negative predictive values for the investigated biomarkers are listed in Table 4.

## 4. Discussion

Within the last two decades, research on the potential useful and new biomarkers in the context of heart failure was undertaken without significant improvement in our diagnostic routine [7,9,10,11,12,15,16,17]. To date, the only guideline-recommended biomarkers for HF are natriuretic peptides and troponin [3]. While their daily use is limited to HF diagnosis, their therapy guiding properties have not yet been proven. Natriuretic peptides are not specifically elevated only in HF. An enhancement can also be due to chronic kidney disease, atrial fibrillation, hypertension, and cardiac valve disease. Those diseases can also cause an elevation of troponin and no parameters clearly indicate heart failure. Therefore, the quest remains for alternative candidates. For our study, we selected the growth differentiation factor-15 (GDF-15), which has previously been describes as a biomarker of oxidative stress in myocardium [18,19,20,21]. Recently, it has been pointed out as a marker is heart failure management and cardiac remodeling [22]. Furthermore, in our study, we investigated the biomarker H-FABP (heart-type fatty acid binding protein), a low molecular weight protein expressed in cardiac myocytes [23]. H-FABP is released in the context of myocardial damage and LV remodeling [14,24]. Likewise, suPAR occupies a direct role during cardiac remodeling; through cleavage of membrane-bound uPAR at the GPI anchor, suPAR is released. Endothelial dysfunction is also associated with elevated suPAR- levels [25,26,27,28]. VCAM-1 (cell adhesion molecule 1) has been described as a biomarker in chronic inflammation and is elevated in ischemic heart failure patients [5,29,30].

We found elevated levels of the circulating biomarkers suPAR, GDF-15, VCAM-1, and H-FABP in patients with heart failure. This result is in accordance with previous studies reporting the expression of those biomarkers in heart failure. The role as a potential predictive biomarker to distinguish between the group of compensated and acute decompensated heart failure was unclear. In our study, we present data indicating for the first time that levels of suPAR, GDF-15, VCAM-1, and H-FABP were significantly increased in the decompensated group in comparison to a group of patients with compensated HF. These findings could reflect ongoing inflammation, cardiac remodeling, and tissue damage in decompensated heart failure.

As serum NT-proBNP is 4-fold higher in decompensated versus compensated HF patients, it is still not possible to differentiate and diagnose decompensated HF patients using this biomarker alone. Adding potential new biomarkers, such as our herein tested candidates, might indeed synergistically improve the sensitivity and specificity for the future diagnosis of congested heart failure.

While H-FABP was initially considered due to its role in myocardial injury, its strong correlation with CRP, BMI, and creatinine led us to deprioritize it in our multivariate analysis.

Our study group with a total of 146 patients is limited in number and was obtained from one center. Most of the patients were undergoing recommended treatment for heart failure (Table 1). As is different from the recommendations of the 2021 published ESC guidelines for the management of heart failure, only 1.9% of patients with compensated heart failure and 7.3% of acute decompensate HF patients received SGLT-2 inhibitors. This could be due to the fact that patient recruitment was performed before the introduction of the new guidelines. Moreover, we were able to determine a significant difference in BMI of the compensated and acute decompensated HF patients, which could have an impact on chronic inflammation that is able to drive disease progress. It is also likely that edema leads to enhanced body weight by fluid retention and hereby elevated BMI in the decompensated HF group. Since we can only speculate on the impact of those findings and those could also be artificial due to the small clinical cohort, further studies are required to investigate the role of SGLT-2 inhibitors and cardiovascular risk factors on HF worsening.

Although the mechanisms leading to oxidative stress in the myocardium, cellular damage, and chronic inflammation in heart failure are not fully understood, the biomarkers could be either a mediator of acute heart failure or a by-product of cellular damage, allowing us to estimate disease progress.

From a clinical perspective, finding new diagnostic tools in routinely performable biomarker assays would allow for the quick detection of patients at risk of severe acute decompensation heart failure and would bring the potential to reduce mortality in this highly vulnerable patient group.

Albeit we evaluated several circulating biomarkers for their predictive value in acute decompensated heart failure, it remains important to consider their potential impact in our small study group obtained from one single center in Germany. The absence of a control arm limits interpretation, since it is likely that the natural course of worsening of HF will have an impact on the baseline values. The cross-sectional design of the study limits informative value regarding the changes in biomarker concentrations over time. Moreover, the unclear and often overlapping definition of compensated and acute decompensated HF are a challenge when interpreting clinical studies. Nonetheless, our study points out a significant increase in the biomarkers GDF-15, suPAR, and VCAM in patients in the status of acute decompensated heart failure. However, further studies are required to investigate potential prognostic parameters, such as biomarkers. Large, multicenter-trials or studies with a longitudinal design are needed to validate our detected biomarkers GDF-15, suPAR, and VCAM or evaluate any other biomarkers in their predictive value. Furthermore, novel biomarkers need to be evaluated for potential confounders to make sure that they are specific, sensitive, and useful parameters. By unveiling novel biomarkers that are able to effectively distinguish between patients at risk for acute decompensated heart failure or patients with stable disease conditions, we access easy tools for our daily clinical practice. Moreover, a faster diagnostic track will most likely lead to an earlier and faster therapy initiation. Patients could benefit from a quicker diagnostic workup at the emergency department. This would allow earlier and better stabilization of the patients’ condition and could lead to a reduction in mortality due to acute HF. Additionally, novel biomarkers could be a useful tool in managing and guiding the therapy and evaluation of therapy success.

## 5. Conclusions

In summary, we were able to point out a significant elevation of the circulating biomarkers GDF-15, suPAR, and VCAM-1 in acute decompensated heart failure. The elevation could reflect ongoing and reinforced inflammation, cardiac remodeling, and tissue damage during acute disease worsening. As our work suggests, further research is required to evaluate and establish predictive and clinically useful biomarkers in our daily routine. These biomarkers could be cost-effective and novel tools in our daily interaction with HF patients. They could be a useful tool in guiding the therapy and in the evaluation of therapy success. Finally, novel biomarkers, which will allow us to distinguish between decompensated disease or patients at risk for decompensation and stable disease, are needed to reduce HF mortality through the early detection of patients at risk.

## Figures and Tables

**Table 1:** Baseline characteristics from investigated patients.

Basics	Compensated (*n* = 105)	Decompensated (*n* = 41)	*p*-Value
	Median (IQR)/%	Median (IQR)/%	
Gender (male)	72.38%	70.73%	0.843
Age (years)	64 (17)	69 (23.5)	0.272
BMI (kg/m^2^)	25.9 (6.1)	30.3 (8.8)	<0.001
medication			
ACE/AT1	70.5%	56.1%	0.116
betablockers	89.5%	78.0%	0.116
aldosterone-antagonist	61.9%	36.6%	0.006
loop diuretics	60.0%	75.6	0.064
SGLT-2-inhibitor	1.9%	7.3%	0.217
platelet inhibitor	37.1%	14.6%	0.003
thiazide diuretics	6.7%	9.8%	0.528
angiotension receptor-neprilysin inhibitor	21.9%	14.6%	0.295
labor values			
Hb (g/dL)	14 (2.8)	13.4 (2.4)	0.379
NTproBNP (pg/mL)	1207 (1900)	3708.50 (6885)	<0.001
HbA1C (%)	5.5 (0.7)	6.050 (1.6)	0.055
LDL (mg/dL)	86 (44)	86.50 (36)	0.445
Creatinine mg/dL	1.0 (0.4)	1.25 (0.7)	0.004
NYHA-stadium			
NYHA I	11.4%		0.026
NYHA II	45.7%	9.8%	<0.001
NYHA III	40.0%	63.4%	0.008
NYHA III *	35.7%	57.6%	<0.001
NYHA IV	2.9%	22.0%	0.007
medical history			
edema	11.4%	61.0%	<0.001
pleural effusion	1.9%	26.8%	0.001
angina pectoris	9.5%	19.5%	0.157
arterial hypertension	59.0%	68.3%	0.297
family history	30.5%	24.4%	0.469
dyslipidemia	41.9%	31.7%	0.250
diabetes mellitus	25.7%	43.9%	0.046
smoking history	48.6%	48.8%	0.982
syncope	2.9%	7.3%	0.159
cardiac etiology			
ischemic	39.0%	41.4%	0.823
not ischemic	60.9%	58.5%	0.872
ejection fraction			
LVEF (%)	39 (18.25)	35 (22)	0.205
HFmrEF	29.5%	21.9%	0.157
HFrEF	78.1%	75.6%	0.564
HFpEF	15.2%	19.5%	0.581

BMI: Body Mass Index; ACE: Angiotensin-Converting-Enzyme-Inhibitor; AT1: Angiotensin 1-Receptor-Inhibitor; SGLT-2-Inhibitor: Sodium-Glucose-Linked-Transporter-2-Inhibitor; NTproBNP: N-Terminal Pro-B-Type Natriuretic Peptide; NYHA: New York Heart Association; Hb: Hemoglobin; LDL: Low-Desity-Lipoprotein; LVEF: Left-Venticular-Ejection-Fraction. HFmrEF: heart failure with mildly reduced ejection fraction; HFrEF: heart failure with reduced ejection fraction; HFpEF: heart failure with preserved ejection fraction; IQR: interquatile range = median of upper half of values − median of lower half of values. NYHA III * = NYHA III + sign of decompensation.

**Table 2:** Correlation after univariate analysis of baseline characteristics and biomarkers.

	suPAR	VCAM-1	GDF-15	H-FABP
*p*-value	0.0015	0.0015	0.0352	0.3401

**Table 3:** Multivariate regression. Correlation after multivariate analysis with confounders BMI, CRP, creatinine, and diabetes mellitus.

	suPAR	*p*-Value	VCAM-1	*p*-Value	GDF-15	*p*-Value	H-FABP	*p*-Value
	r		r		r		r	
EF	0.170	0.056	0.170	0.056	0.130	0.144	0.204	0.022
IVSDd (mm)	0.423	0.000	0.424	0.000	0.225	0.011	0.206	0.020
BMI kg/m^2^	0.232	0.007	0.232	0.007	0.113	0.191	0.004	0.961
Age	0.311	0.000	0.311	0.000	0.495	0.000	0.366	0.000
NTproBNP (pg/mL)	0.452	0.000	0.452	0.000	0.591	0.000	0.453	0.000
Creatinine (mg/dL)	0.480	0.000	0.480	0.000	0.559	0.000	0.479	0.000
CRP	0.464	0.000	0.464	0.000	0.443	0.000	0.320	0.000

**Table 4:** Rates of sensibility, specificity, and positive and negative predictive values for all tested biomarkers in acute decompensated and compensated heart failure patients.

	Sensitivity	Specificity	PPV	NPV
GDF-15	85.70%	51.30%	58.80%	81.50%
H-FABP	69.40%	74.40%	52.70%	87.10%
suPAR	87.80%	71.80%	70.00%	88.60%
VCAM-1	87.80%	71.80%	70.00%	88.60%

## Data Availability

Please contact the corresponding authors in the case of any request on the data published here.

## Supplementary Materials

The following supporting information can be downloaded at: https://www.mdpi.com/article/10.3390/jcm13226866/s1, Table S1: *p*-values of biomarkers after univariate regression of aldosterone antagonist, SGLT-2-inhibitor and platelet inhibitor.
