# Supplementary material for: Novel Biomarkers as Potential Predictors of Decompensated Advanced Chronic Heart Failure—Single Center Study
Source: J Clin Med. 2024 Nov 14;13(22):6866. doi: 10.3390/jcm13226866 (PMC11594948; doi:10.3390/jcm13226866)
Supplement: Supplementary file 1 [file jcm-13-06866-s001.zip › jcm-3310830-supplementary.pdf]

Supplementary Material Table S1: *p*-values of biomarkers after univariate regression of aldosterone antagonist, SGLT-2-inhibitor and platelet inhibitor.

	***p*-value**			
	**suPAR**	**H-FABP**	**GDF-15**	**VCAM-1**
Aldosterone antagonist	0.080	0.134	0.158	0.080
SGLT-2-inhibitor	0.411	0.738	0.920	0.411
Platelet inhibitor	0.107	0.082	0.239	0.107
